# Dynamic Evolution of Land Use/Land Cover and Its Socioeconomic Driving Forces in Wuhan, China

**DOI:** 10.3390/ijerph20043316

**Published:** 2023-02-14

**Authors:** Qijiao Xie, Yidi Han, Liming Zhang, Zhong Han

**Affiliations:** Faculty of Resources and Environmental Science, Hubei University, Wuhan 430062, China

**Keywords:** land use/land cover, spatiotemporal transition, driving force, socioeconomic factor

## Abstract

Human activities are considered as the main driving forces of land use/land cover (LULC) variation at city scales. Monitoring the dynamic variation of LULC and its socioeconomic driving forces helps to reveal the response of LULC change to human activities and land use policies. However, this issue remains poorly understood. In this study, the spatiotemporal transitions among different LULC types during nearly three decades in Wuhan, China, were modeled in detail using the transfer matrix method. Ten socioeconomic factors indicating the population level, economic condition and social development were selected to quantitatively explain LULC variation. Some typical policies were discussed for the LULC transitions. The results showed that construction land was detected to continuously increase, with the fastest change rate of 560.48% during the 29-year period. Farmland area significantly declined by 1855 km^2^, decreasing by 31.21%, contributing to 86.14% of the area increase in construction lands. To some extent, the net area increase in construction land was at the expense of farmland area. All 10 indicators considered in this study were positively correlated with the construction land area (R^2^ of 0.783~0.970) and negatively correlated with farmland area (R^2^ of 0.861~0.979). In general, social and economic development contributed considerably to urban expansion and cultivated land loss. The largest contributors were non-agricultural population and economic conditions (secondary industry output, primary industry output and local revenues). Governmental guidance and behavior were considered the original impetus for LULC transition, while the impact of land use policies and human activities on LULC transitions varied across the subperiods. These findings provide decision-making support for appropriate urban planning and efficient land use management.

## 1. Introduction

Land use/land cover (LULC) is the link between humans and nature [1,2,3]. Land use transition often occurs as a result of changes in economic and social development [4,5]. The transforming direction, speed and degree in different stages are significantly affected by the government’s economic development goals and land use policies [6,7,8,9]. In the stage of traditional agriculture, social development highly depends on the output of agricultural products. The government guarantees the supply of cultivated land by encouraging land reclamation and protecting farmland [10,11]. With the development of modern agricultural technology and the enhancement of farmers’ environmental protection awareness, the demand for cultivated land expansion has declined. The government guides and implements environmentally friendly land use to meet the needs of economic development by increasing capital and technology investment rather than farmland expansion [12,13,14]. Since the 20th century, urbanization and industrialization have greatly promoted social and economic development. The government’s economic policies act on the land use process, resulting in the rapid increase in construction land and a sharp decrease in natural resources [15,16,17,18]. However, economic development and urban expansion also lead to a series of ecological and environmental problems, such as air quality deterioration, water shortage, land degradation and global warming, with the original LULC patterns significantly altered [19,20,21,22]. The government can influence the process of ecosystem reorganization and renewal through policy intervention. Some resource protection policies are needed to combat environmental degradation by restraining construction land expansion and strengthening natural resource protection [9,23,24].

Land use transition is the result of the regional conditions and economic and social development associated with government policies. Monitoring dynamic variations in the LULC and their main driving forces not only offers a vital means to reveal the response of LULC change to land use policies and human activities but also provides decision-making support for efficient land use management [2,25,26]. However, land use policies interact with each other and are difficult to monitor directly [27,28,29]. Social and economic development conditions are closely related to land use policies, which are implemented through human activities such as urbanization [30,31], cultivation [32], plantation [33,34] and deforestation [9]. Detecting the impact of socioeconomic factors on LULC variation provides sound evidence to better understand the relationship between human activities and environmental changes and to scientifically adjust land use policies. However, this issue remains poorly understood [25,35].

Much attention has been paid to the spatiotemporal variations in single LULC types, such as urban land [30], farmland [32], greening land [28] and water bodies [36], to develop appropriate land use management and policies [37]. With the drastic increase in urban population, the profound need for construction land increased accordingly, especially in economically developed areas [38,39,40]. Close links have generally been detected between urban expansion and socioeconomic factors such as urbanization and industrialization level, real estate development, population growth and related policy implementation [37,41,42]. Due to urban expansion, vast amounts of cropland have been replaced by buildings, roads, parking lots and squares [32,43,44]. The dynamic change of cultivated land was mainly influenced by the combination of economy, population, nature and location-based driving forces [45]. Specifically, the decrease in farmland area is mainly attributed to local economic development and population increase [17,46], while the pattern variation in arable land is influenced by geographic position and natural conditions [11,47]. The widespread loss of agricultural land area affects the local climate, worsens the ecological environment and reduces the output of regional crops, which threatens the survival and development of humanity [48]. To meet the demand of food production and urban expansion induced by population growth, a lot of vegetation areas and water bodies have been encroached or occupied [10,11,36]. Land use policies of deforestation for food production and reforestation for environmental protection [8,23,45] have been irregularly implemented in different regions and periods.

The abovementioned studies focused on investigating construction land, water bodies, forest or cropland change in different regions and periods, which provided scientific and powerful support for rational land use. The impact of driving forces on land use structures and patterns is complex and multidirectional [2,49,50]. For a given land use type, its variation involves the transfer process from other types and to other types and varies over time and space [15,51]. A systematic analysis of the cross transformation of different land use types can help to identify the direction and extent of transfer in detail and to explore the specific driving forces of LULC change more scientifically. However, previous studies paid little attention to the dynamic transformation among different LULC types [2,7]. Few studies involved potential factors such as geographical condition and related policy factors in observing the main driving forces [13,52].

Therefore, this study aims to describe the dynamic evolution of different LULC types and seek the main driving forces from a socioeconomic perspective. The main objectives are: (1) to monitor LULC (namely farmland, greening land, water bodies and construction land) changes from 1987 to 2016 in Wuhan, China; (2) detect the spatiotemporal transitions among different LULC types during the periods of 1987–1996, 1996–2007 and 2007–2016; (3) investigate the socioeconomic development and its impact on LULC change during the study periods to explore the main socioeconomic driving forces; and (4) discuss how human activities associated with land use policies and economic development policies influence land transitions in different subperiods.

## 2. Materials and Methods

### 2.1. Study Area

**Figure 1:**
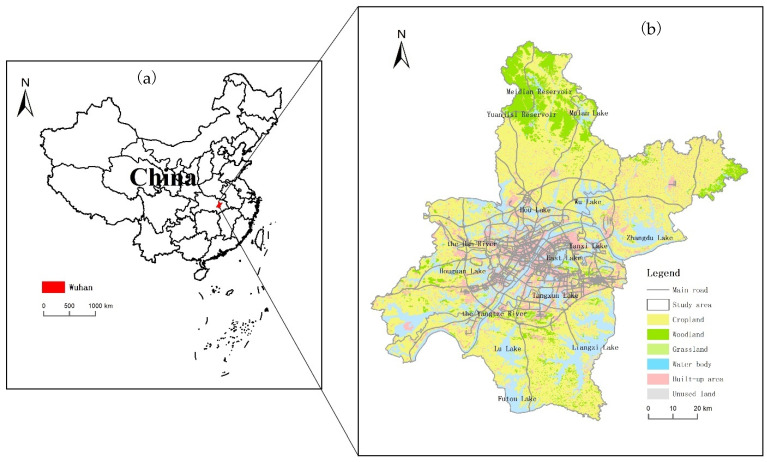
Study area description: (**a**) location of Wuhan in China and (**b**) land use of Wuhan.

This study was carried out in Wuhan, the capital of Hubei province in China. Wuhan is the only megacity in central China and one of the most important national transportation hubs in the country (Figure 1a). It covers an area of more than 8500 km^2^, including 13 administrative districts. With a history of more than 3500 years, Wuhan has a population of over 11 million. It is divided into three parts, namely Wuchang, Hankou and Hanyang, by the Yangtze River (the third largest river in the world) and its largest tributary, the Han River (Figure 1b).

Wuhan is situated at 29°58′~31°22′ N, 113°41′~115°05′ E. It is located in Jianghan Plain, with the plain area mainly concentrated in the middle and surrounded by rolling hills and mounds. More than 80% of the total area is covered by plains, and the altitude is below 50 m in most areas of Wuhan. Wuhan has a monsoon (humid) climate in the north subtropical zone, with cold winters and hot summers. It is characterized by distinct seasons, sufficient heat and abundant rainfall. The average annual temperature ranges from 15.8 °C to 17.5 °C, with the extreme high temperature of 41.3 °C occurring on 10 August 1934 and the extreme low temperature of −18.1 °C on 30 January 1977. The annual precipitation is 1150–1450 mm, about 40% of which is concentrated from June to August. The annual frost-free period is generally 211~272 days, and the total annual sunshine hours are 1810~2100 h.

### 2.2. LULC Classification

In this study, three Landsat-5 TM images acquired on 26 September 1987, 4 October 1996 and 10 April 2007 and one Landsat-8 TM image acquired on 23 July 2016 were used to map the LULC patterns. They were downloaded from the Chinese geospatial data cloud website (http://www.gscloud.cn/, accessed on 21 September 2018) with cloud coverage of less than 10%. All the Landsat images were rectified and georeferenced to the Universal Transverse Mercator (UTM) projection system (spheroid WGS84, datum WGS84, zone 50) before being interpreted.

A supervised classification method using a maximum likelihood algorithm was employed to classify all considered satellite images in ENVI software. All the images were classified into four major LULC categories, which were closely related human activities [28,30,32,36]: (1) farmland, which refers to arable agriculture land, including paddy fields, dry land and vegetable fields but not orchards; (2) greening land, which refers to vegetated areas, including forest land, grassland, orchards and urban green spaces but not cropland; (3) water bodies, including rivers (e.g., the Yangtze River and the Han River), lakes, ponds and reservoirs; (4) construction land, which refers to developed areas, including industrial areas, commercial regions, residences, transportation facilities and other infrastructure sites. For each LULC type, 250 samples were randomly selected to check the classification accuracy. The overall classification accuracies and kappa coefficients for 1987, 1996, 2007 and 2016 were 87.54%, 92.39%, 89.13% and 94.89% and 0.83, 0.90, 0.86 and 0.89, respectively.

### 2.3. Land Use Transfer Matrix

A transition matrix is usually use to describe the extent and direction of land transitions among different LULC types. The transition matrix plays an important role in clarifying the detailed transition information and monitoring of LULC change [26]. In this study, transfer matrices for the periods of 1987–1996, 1996–2007, 2007–2016 and 1987–2016 were produced as follows:

(1) $$S_{ij}=\left[\begin{array}{ccccc} S_{11} & S_{12} & S_{13} & \cdots & S_{1n}\\ S_{21} & S_{22} & S_{23} & \cdots & S_{2n}\\ S_{31} & S_{32} & S_{33} & \cdots & S_{3n}\\ \cdots & \cdots & \cdots & \cdots & \cdots \\ S_{n1} & S_{n2} & S_{n3} & \cdots & S_{\mathrm{nn}} \end{array}\right]$$

where *S* represents the area; *n* is the number of land use types; and *i* and *j* (*i*, *j* = 1, 2, ···, *n*) are the land use types before and after the transition, respectively. The first column refers to the conversion area from other land use types to the first type, and the first row indicates the conversion area from the first land use type to other types. The other rows and columns can be interpreted in the same way.

### 2.4. Modeling the Driving Mechanism

Anthropogenic driving forces such as population growth, economic increase and accelerated social development were confirmed as the dominant drivers of LULC changes on a local scale [5,6]. Referring to the previous research results [15,26,47], we selected the following 10 socioeconomic factors as independent variables: total population (X1), non-agricultural population (X2), gross domestic product (X3), primary industry output (X4), secondary industry output (X5), tertiary industry output (X6), fixed asset investments (X7), local revenues (X8), per capita road area (X9) and the motor vehicles (X10). They were considered as the potential driving forces leading to LULC change in this study, indicating the population level (X1, X2), economic condition (X3–X8), and social development (X9, X10) in Wuhan. The area of LULC type (Y) was the dependent variable. The regression equation was modeled through the step regression method as:

(2) $$Y=b_{0}+b_{1}X_{1}+b_{2}X_{2}+b_{3}X_{3}+\cdots +b_{i}X_{i}$$

where Y is the area of a certain LULC type, X*_i_* (*i* = 1…p) is the indicator mentioned above, b_0_ is the constant term of the equation and b*_i_* (*i* = 1…p) is the regression coefficient.

All these variables were primarily obtained from the Wuhan Statistical Yearbook (Data for and after 2009 were obtained from http://tjj.wuhan.gov.cn/tjfw/tjnj/ (accessed on 22 November 2019), and data before 2009 were available from the Wuhan Bureau of Statistics based on the paper version) and supplemented by data from the China Urban-Rural Construction Statistical Yearbook and the National Bureau of Statistic official website (http://www.stats.gov.cn/tjsj/ndsj/, accessed on 28 November 2019). The land use policies and socioeconomic policies involved in this study were mainly referenced from the official websites of the People’s Government of Hubei Province (http://www.hubei.gov.cn/, accessed on 23 May 2020), the Wuhan Municipal People’s Government (http://www.wuhan.gov.cn/, accessed on 23 May 2020) and the Wuhan Natural Resources and Planning Bureau (http://gtghj.wuhan.gov.cn/, accessed on 24 May 2020).

## 3. Results

### 3.1. Spatial Distribution and Variation of LULC

**Figure 2:**
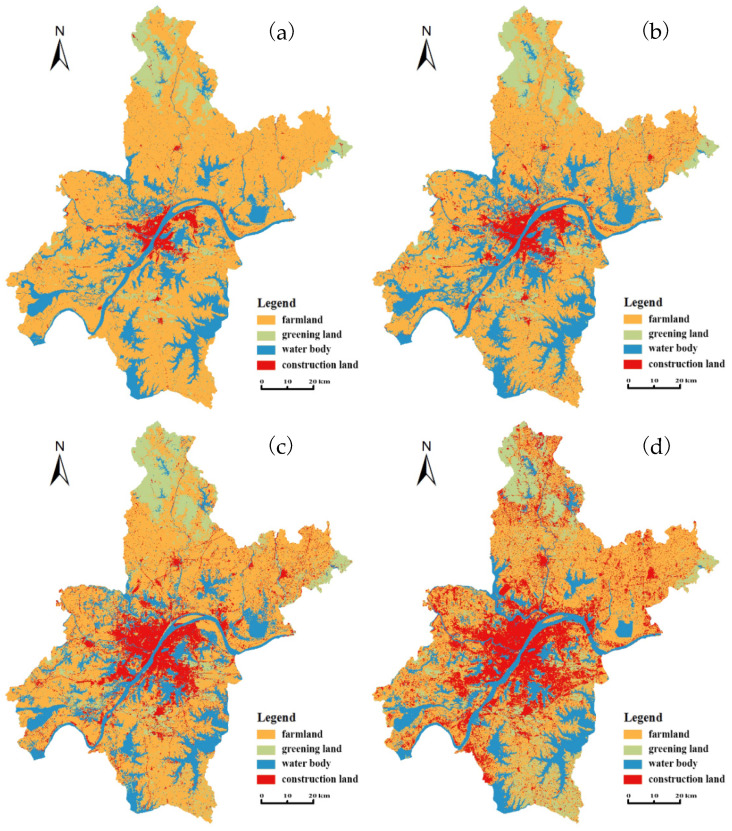
LULC distribution maps of Wuhan for (**a**) 1987, (**b**) 1996, (**c**) 2007 and (**d**) 2016.

Figure 2 displays the LULC spatial distribution maps of Wuhan for 1987, 1996, 2007 and 2016. They share similar spatial patterns, i.e., construction lands are mainly located at the city core and surrounded by a large area of water bodies and farmland. Greening land is concentrated in the north and northeast forests and scattered in urban green spaces in the study area. Farmland was the most dominant LULC type in the study years, covering 69.26%, 60.73%, 53.30% and 47.64% of the study area in 1987, 1996, 2007 and 2016, respectively (Table 1). Water bodies was the second most dominant LULC type during the study periods, except in 2016, covering 18.96%, 22.48%, 17.74% and 16.77% in 1987, 1996, 2007 and 2016, respectively. Construction land had the least coverage in 1987 (3.39%), 1996 (6.28%) and 2007 (13.65%), while in 2016, construction land area exceeded water area, covering 22.40% of the study area.

Table 1 also records the specific information on LULC area change and its change rate in different study periods. Construction land was detected to continuously increase during the entire study period, with the area increasing from 291 km^2^ in 1987 to 1922 km^2^ in 2016. During the 29-year period, construction land expanded by 560.48%, making it the fastest-growing LULC type. As shown in Figure 2, areal extent colored in red significantly expanded from 1987 to 2016, indicating that construction land spread out along the Yangtze River and the major roads. The coverage area of farmland continuously decreased from 1987 to 2016, decreasing by 31.21%. During this period, 1855 km^2^ of farmland was lost, representing largest area loss. In general, the area covered by forests and urban green spaces increased by 57.22%, representing an increase of 412 km^2^ from 1987 to 2016. The water area was contracted by 11.56%, declining from 1627 km^2^ in 1987 to 1439 km^2^ in 2016.

Although the area changes of the four LULC types varied in different study periods, their change rates showed similar regular patterns. Almost all LULC types changed the fastest during period of 1996–2007, with changing rates of −12.22%, 45.68%, −21.10% and 117.25% for farmland, greening land, water bodies and construction land, respectively. The next most rapid land type changes occurred during the period of 1987–1996 (25.28%, 18.56% and 85.22%) and 2007–2016 (−13.85%, −5.45% and 64.13%). In contrast to the continuous decrease in farmland area and the continuous increase in construction land area in the entire study period, the area change trends of greening land and water bodies were more complex. Greening land experienced an area increase during both 1987–1996 and 1996–2007 and decreased during 2007–2016. Water area expanded during 1987–1996 but shrunk during both 1996–2007 and 2007–2016.

### 3.2. Spatiotemporal Transitions of LULC from 1987 to 2016

**Figure 3:**
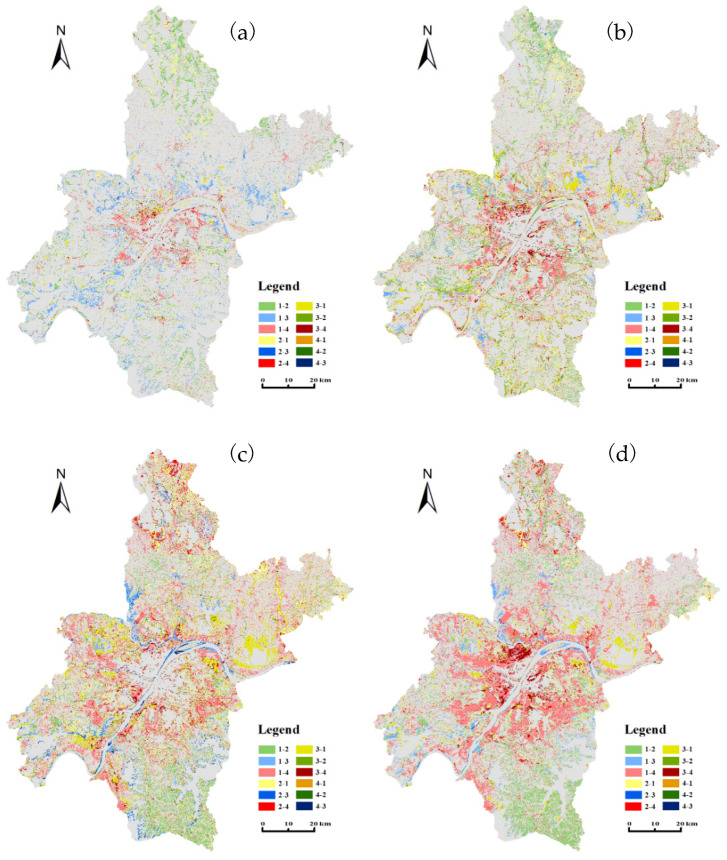
Spatiotemporal transitions among different LULC types during (**a**) 1987–1996, (**b**) 1996–2007, (**c**) 2007–2016 and (**d**) 1987–2016. Note: 1, 2, 3 and 4 represents farmland, greening land, water bodies and construction land, respectively, and “-” represents the transformation process among LULC types. For example, “1–4” expresses the conversion from farmland to construction land, “2–4” expresses the conversion from greening land to construction land and the rest of the legend can be interpreted in the same way.

To better understand the area and space conversions among different land types, the LULC transfer matrix was modeled. Table 2 lists the conversion areas between farmland, greening land, water bodies and construction land. Figure 3 illustrates the spatial extents of the land transitions among different LULC types from 1987 to 2016. Area and space transitions significantly varied across land transition types and the three subperiods.

Between 1987 and 1996, the three main land conversions were from farmland to water bodies (449 km^2^), from farmland to greening land (326 km^2^) and from farmland to construction land (272 km^2^). Farmland was the major contributor to the inward transfer of water bodies (93.93%), greening land (91.06%) and construction land (86.08%). As shown in Figure 3a, the expansion of construction land mainly occurred in inner-city areas, along the main traffic roads. The area increase in water bodies primarily occurred in the urban fringe, and the greening extent was mainly developed in the suburbs.

Between 1996 and 2007, the most common land conversions were from farmland to construction land (578 km^2^) and greening land (525 km^2^), accounting for 44.50% and 40.42% of the total loss of farmland, respectively, followed by the transition of water bodies to farmland (386 km^2^), contributing 58.31% to the inward transfer of farmland. Figure 3b indicates that construction land expanded outward around the inner city, with the extent mainly extending from the first ring road to the second ring road. The transition from farmland to greening land was scattered outside the built-up areas or lined along large rivers and wide traffic roads.

Between 2007 and 2016, the most dominant land transition was from farmland to construction land, with a transferred area of 906 km^2^, accounting for 58.26% of the outward transfer of farmland and contributing to 78.37% of the inward transfer of construction land. The urban extent spread out along the third ring road, and construction land expanded to the suburbs, as shown in Figure 3c. The two-way transformation between farmland and greening land was almost equal, with areas of 452 km^2^ from farmland to greening land and 446 km^2^ from greening land to farmland.

During the entire period of 1987–2016, 2311 km^2^ of farmland was transformed into other land types, of which 62.66% was transferred into construction land, 27.95% into greening land and 9.39% into water bodies. Construction land had the largest area of inward transfer from other land types, with a value of 1681 km^2^. Proportions of 86.14%, 7.67% and 6.19% of construction land were converted from farmland, water bodies and greening land, respectively. In general, the net area increase in construction land occurred at the expense of farmland area. Figure 3d illustrates the extent and direction of transformation among different LULC types across the entire studied period. Some farmland around large water bodies was occupied by construction land, and such lands in the southeast of the study area were partly used for greening land.

### 3.3. Driving Mechanism

#### 3.3.1. Temporal Variation Trends of Socioeconomic Conditions

**Figure 4:**
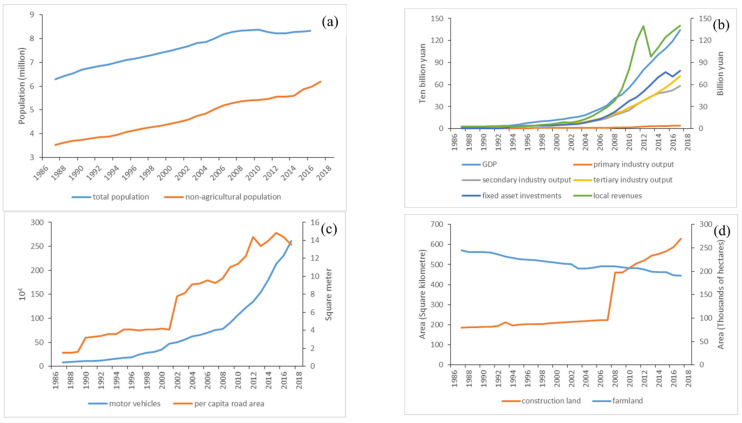
Temporal variations of (**a**) population, (**b**) economical conditions, (**c**) social development and (**d**) areas of farmland and construction land from 1987 to 2016 in Wuhan.

Extensive LULC change and its spatial variation were considered as a result of frequent human activities. Increased social demand and rapid economic development were the main driving forces for LULC transition. Figure 4 shows the changing trends of social and economic conditions in time series during the entire study period. Figure 4a indicates that both total population and non-agricultural population continuously increased from 1987 to 2016 in the study area. The non-agricultural population was increased by 69.89 from 3.52 million in 1987 to 5.98 million in 2016. The economic indicators except the primary industry output express similar changing trends during the entire study period. Gross domestic product (GDP), fixed asset investments, local revenue, secondary industry output and tertiary industry output experienced steady growth from 1987 to 2004 and a rapid increase from 2005 to 2016 (Figure 4b), while the primary industry output maintained relatively stable growth between 1987 and 2016. Since 2005, the growth of social demand has accelerated significantly, especially for fixed asset investments. As shown in Figure 4c, motor vehicles and per capita road area exhibited increasing trends from 1987 to 2016. Figure 4d shows the area changing trends of farmland and construction land during the three decades of the study period. New lifestyles and needs associated with urbanization resulted in the decrease in farmland area and the increase in construction land extent. In general, the area increase in construction land occurred at the expense of the farmland area.

#### 3.3.2. Impact of Socioeconomic Factors on Urban Expansion

**Figure 5:**
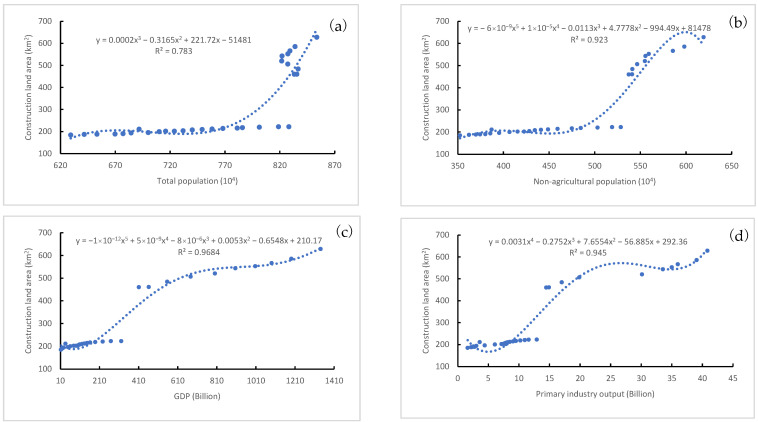

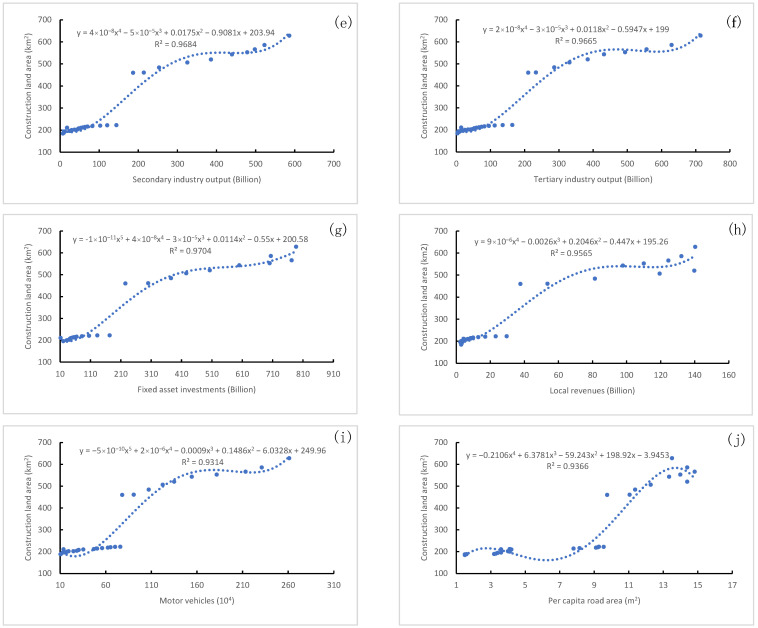
Relationships between construction land area and (**a**) total population, (**b**) non-agricultural population, (**c**) GDP, (**d**) primary industry output, (**e**) secondary industry output, (**f**) tertiary industry output, (**g**) fixed asset investments, (**h**) local revenues, (**i**) motor vehicles and (**j**) per capita road area during 1987–2016 in Wuhan.

In order to fully clarify the impacts of social and economic conditions on LULC transitions, we modeled the relationships between the selected socioeconomic indices and construction land area, as well as farmland area. Figure 5 illustrates the regression curves between all 10 driver indicators and construction land area. The results indicate that construction land area was positively correlated with all indicators considered in this study, with R^2^ values ranging from 0.783 to 0.9704. In general, social and economic development contributed significantly to urban expansion. Both total population (Figure 5a) and non-agricultural population (Figure 5b) increased at the same rate as urban expansion from 1987 to 2007, showing a linear regression relationship between them. However, the area of construction land has increased rapidly since 2008, while the total population has fluctuated obviously since 2011. The imbalance in variation between these two variables weakened the correlations between construction land area and population. During 2007–2016, urban expansion was no longer constrained by total population and non-agricultural population.

Six indicators related to economic conditions, i.e., GDP (Figure 5c), primary industry output (Figure 5d), secondary industry output (Figure 5e), tertiary industry output (Figure 5f), fixed asset investments (Figure 5g) and local revenue (Figure 5h), had a similar impact on the increase in construction land area. The turning point in the correlations between the economic indicators and construction land area was also marked in 2007. Economic development and urban construction increased at a coordinated rate between 1987 and 2007. During 2007–2016, the increase rate of construction land was significantly faster than that of economic development. The same is true of the collaborative change between construction land area and the number of motor vehicles (Figure 5i). Due to the unstable growth of per capita road area, its impact on the construction land area increase was relatively complex (Figure 5j).

#### 3.3.3. Impact of Socioeconomic Factors on Farmland Area Change

**Figure 6:**
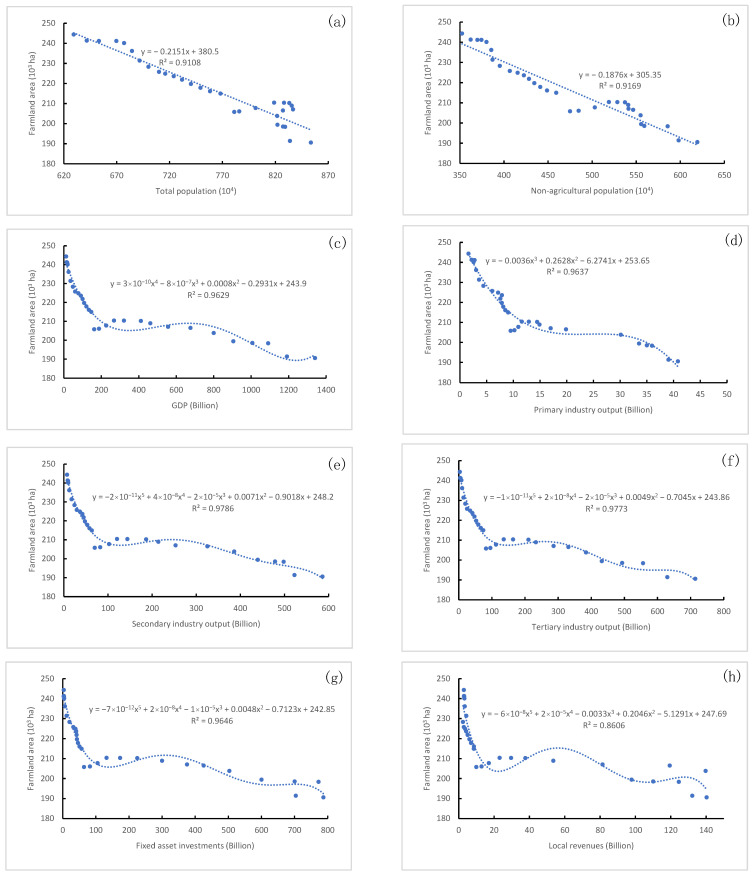

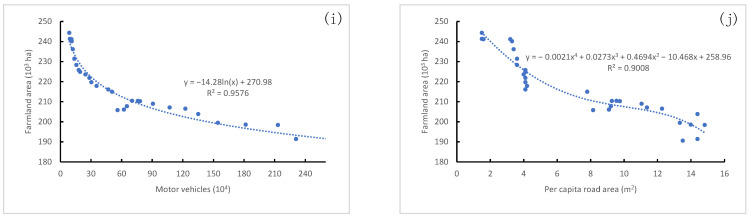
Relationships between farmland area and (**a**) total population, (**b**) non-agricultural population, (**c**) GDP, (**d**) primary industry output, (**e**) secondary industry output, (**f**) tertiary industry output, (**g**) fixed asset investments, (**h**) local revenues, (**i**) motor vehicles and (**j**) per capita road area during 1987–2016 in Wuhan.

Figure 6 shows the regression curves between all 10 driver indicators and farmland area. The results indicate that farmland area was negatively correlated with all indicators considered in this study, with R^2^ values ranging from 0.861 to 0.979. These negative correlations between socioeconomic factors and farmland area indicate that farmland area decreased with the improvement of economic and social conditions between 1987 and 2016. To some extent, economic and social development occurred at the expense of farmland area. Both total population (Figure 6a) and non-agricultural population (Figure 6b) were detected to have linear negative correlations with farmland area, with R^2^ values of 0.911 and 0.917, respectively. Correlations between farmland area and seven investigated indicators, i.e., GDP (Figure 6c), primary industry output (Figure 6d), secondary industry output (Figure 6e), tertiary industry output (Figure 6f), fixed asset investments (Figure 6g), local revenue (Figure 6h) and the number of motor vehicles (Figure 6i), expressed similar variation trends during the entire study period. The contribution of social and economic development to the reduction in farmland area between 1987 and 2007 was significantly greater than that between 2007 and 2016. After 2007, economic development continued to accelerate, while the decreasing rate of farmland area slowed down. Figure 6j shows that the increase in per capita road area resulted in a reduction in farmland area, with a determination coefficient of 0.901.

#### 3.3.4. The Main Driving Forces for Area Changes in Construction Land and Farmland

Economic development was considered the internal driving force of the area increase in construction land and area decrease in farmland. As Table 3 shows, construction land area was significantly related to all considered social and economic indices, with the correlation coefficients ranging from 0.741 to 0.957. Significant negative correlations were detected between farmland area and the investigated indicators, with correlation coefficients ranging between 0.734 and 0.953. In addition, significant correlations existed among different socioeconomic variables (Table 3). For example, GDP value was found to be highly correlated with almost all the tested economic indices. The correlation coefficients with primary industry output, secondary industry output, tertiary industry output, fixed asset investment and local revenue were 0.989, 0.999, 0.999, 0.995 and 0.963, respectively. The impact of the socioeconomic index on land area change was not independent and interacted with other related indices.

To investigate the main driving factors, multiple stepwise regressions between construction land area, farmland area and the tested socioeconomic variables were established. Table 4 records the related standardized regression equations and general equations. The standardized regression equation between construction land area and the variables reveals that secondary industry output and primary industry output were the two largest contributors to the increase in the construction land area. These two variables can explain 94.90% of the area change in construction land during the whole studied period. Primary industry output was detected to negatively influence the area variation of construction land, meaning that the urban area expanded as primary industry output decreased. The latter, to some extent, reflects the area of farmland in the same period. In terms of farmland area change, the main driving forces were non-agricultural population and local revenues. The area of farmland declined when non-agricultural population increased and local revenues decreased, accounting for 92.90% of farmland area change between 1987 and 2016.

## 4. Discussion

### 4.1. Economic Development, Human Activities and LULC Change

The most significant variation was found in construction land and farmland, with the largest changed areas (Table 1) and the widest change spaces (Figure 2). All the potential factors considered in this study were observed to significantly correlate with construction land area and farmland area (Table 3) and have obvious impacts on their area changes (Figure 5 and Figure 6). The most dominant drivers were non-agricultural population and economic conditions (secondary industry output, primary industry output and local revenues) (Table 4), which is basically consistent with previous research results [42,53]. In particular, the growth of urban population was found to contribute the most to LULC change in both this study and related studies [26,54].

Population growth itself does not result in large-scale LULC change, but the requirements for higher quality life caused by population growth do [54]. Due to perfect infrastructures, excellent educational environments and professionalized services, cities have a special attraction to all people, especially residents of the suburbs [30,44]. From 1987 to 2016, the total population in Wuhan increased by 35.64%, while the non-agricultural population increased by 75.72% (Figure 4a). In addition to the natural growth of urban population, the population that migrated from the suburbs contributed more to the increased amount. With the enhanced economic benefits from developed areas, some immigrants purchase additional residences in cities but still retain or even build new houses in their hometowns [53]. This phenomenon is mainly attributed to the Chinese people’s unique concept of “home”. They want a balance between preserving their homeland and seeking more development.

With the drastic increase in urban population, the living spaces in cities became crowded and the living environment was deteriorated [44]. To improve housing conditions and living quality, more infrastructure and an increased land supply are needed in urban areas. Large-scale infrastructure construction promotes the rapid development of secondary industries such as construction, industry and manufacturing. Accordingly, secondary industry output and GDP were greatly increased. In this study, secondary industry output and GDP value in 2016 were 71.79 and 95.60 times that in 1987 (Figure 4b), respectively. The process of urban and infrastructure construction involves a lot of laborers, who are mainly from the suburbs around the city. This, in turn, increases the urban population and living demand. The increasing demand for residential area, commercial land, industrial area and traffic roads led to significant urban land expansion [30].

These newly expanded areas were dominantly distributed around the city center and spread along the main traffic roads (Figure 2). The original farmland was constantly encroached and even occupied. To some extent, urban expansion occurred at the expense of farmland, especially around the city center (Figure 3). In addition, urban construction and economic development attracted many rural workers to the city, resulting in a large among of farmland waste or fallow [44]. Due to the large-scale area loss of farmland during the study period (Table 1), although cultivation technology was greatly improved [55], the primary industry output value experienced slower growth than secondary industry and tertiary industry outputs (Figure 4b). This can partly explain the negative impact of primary industry output on construction land change.

### 4.2. Government Behaviors, Land Use Policies and LULC Transition

Construction land area was observed to continuously increase, and farmland continuously decreased from 1987 to 2016 (Table 1 and Figure 4d). However, the interactions between LULC change and the driving factors varied throughout the study periods. The influence of social and economic factors on LULC change was first strong and then weak. It is noteworthy that inflection points of almost all these interactions were marked in 2007 (Figure 5 and Figure 6). Since 2007, the direction and scope of land use transfer has changed significantly (Figure 3). For example, the outward transfer area of greening land and the inward transfer area of farmland between 2007 and 2016 increased significantly compared with the first two periods (Table 2). This was unexpected and seriously inconsistent with the natural process of urbanization [43]. This meant that the LULC variation and transition might be more affected by urban development plans and economic development policies [1,15]. Government’s guidance and behavior were considered the original impetus for LULC transition [49].

Several government-oriented plans and policies were implemented in the study period and varied across the subperiods. Before 1987, due to the low economic level, people’s primary demand was for food production. For a long time, people performed land reclamation activities from lakes and obtained cropland by destroying mountains. With large areas of forests destroyed and water bodies shrunk, some evident ecological consequences, such as drought, flooding and soil erosion, emerged. To combat the negative impacts, the “Law of the People’s Republic of China on Water and Soil Conservation (1991)” was established [12], stating that reclaimed land should be returned to forests or lakes in a planned way. Subsequently, some farmland was transformed into forest, grassland or water bodies under the compulsion of local government (Table 2 and Figure 3a). However, the implementation efficiency remained limited due to the limitation of food production demand.

In order to prevent the further deterioration of the ecological environment and restore ecosystem functions, the Chinese government ramped up the return of farmland to forest by publishing the “Law of the People’s Republic of China on Land Administration (revised in 1998)” and “Some opinions on further improving the policy and measures of returning farmland to Forests (GF [2002] No.10)” [12]. More farmland was converted to greening land between 1996 and 2007 than in the first period (Table 2 and Figure 3b), and the transfer area of farmland to construction land also increased during this subperiod. This land transition was mainly due to the needs of economic development. Since the “System of Paid-for Use for State-owned Land” was implemented in 1998, a large number of farmland has been transformed into construction land (Table 2). This triggered the rapid development of the real estate industry. In 2000, the Wuhan land exchange market was officially listed for operation, indicating the acceleration of urban land expansion in Wuhan (Figure 3b).

With the acceleration of urbanization, urban expansion was no longer constrained by economic development (Figure 6). Many farmland areas were occupied by spreading urban spaces or used to plant fast-growing trees by migrant laborers (Figure 3c), which seriously threatened regional food production. To guide rational urban expansion, the local government issued the “Work plan for the protection and inspection of prime cropland in Wuhan” (Wuhan Natural Resources and Planning Bureau, 2004), which was not finished until 2016 (People’s Government of Hubei Province). By implementing the policy of prime cropland protection, the inward transfer area of farmland in 1987–2016 increased significantly compared with the previous two subperiods (Table 2). Nevertheless, the expansion of construction land still spread to the suburbs in Wuhan (Figure 3c) due to the delay of the policy effect and the need for economic development.

### 4.3. Limitations and Prospects

In recent years, much attention has been paid to LULC transition in China, as it significantly contributes to environmental changes in inland cities [42,44]. In this study, LULC change and transition, combined with their driving forces in Wuhan, were investigated. The results can help local government to better understand the relationship between LULC variation and socioeconomic development, providing sound evidence for land use management. However, this study is subject to some limitations. First of all, a transfer matrix was generated to illustrate areas and directions of land transition among different LULC types. To simplify the transition process, LULC as classified into only four categories in this study according to the literature, namely farmland, construction land, greening land and water bodies. This simplified classification is beneficial to provide overall information about LULC variation but not enough to explore the details of its driving forces. For example, the dominant driving factors for the conversion of farmland to residential land and industrial land are different, although the latter were both grouped as construction land.

It is generally believed that LULC variation and transformation are affected by both natural and socioeconomic factors [2,42]. Wuhan is dominated by plains with mounds and low hills, and geographical and other natural factors have no significant impact on LULC variation [56]. In our study, all 10 indicators related to population (total population and non-agricultural population), economic development conditions (GDP, primary industry output, secondary industry output, tertiary industry output, local revenues and fixed asset investments) and social level (per capita road area and amount of motor vehicles) were considered as potential driving factors that can effectively explain the LULC change during the study period (Table 4). To some extent, the current LULC change is the inevitable result of social and economic development, and related human activities are concentrated in flat plain areas. However, human activities related to both urban expansion and ecosystem protection will gradually spread to mountain areas. The combined effect of natural and socioeconomic forces on LULC variation should be considered in future research.

Like many previous studies [26,53,56], socioeconomic factors were considered to detect their driving mechanism with respect to LULC change in this study. In addition, government behavior and related policies were introduced in discussing the LULC transitions (Section 4.2) because they were defined as determinants of land transition directions among different LULC types [15]. The impact of land use policies on LULC transitions varied across time and space [13,52]. This study confirmed that LULC transitions and its main driving forces can be significantly affected by different government-induced behaviors in different subperiods. However, the specific driving mechanism was still unclear. Quantification of the relationships between land transition and economic development and land use policies in different subperiods and subzones is needed in the future.

## 5. Conclusions

Spatiotemporal transitions among different LULC types such as farmland, greening land, water bodies and construction land from 1987 to 2016 were examined in Wuhan, China. During the 29-year period, construction land area was detected to continuously increase, with the fastest change rate of 560.48%. The increase of 1631 km^2^ was attributed to an inward transfer area of 1681 km^2^ from other land types and an outward transfer area of 50 km^2^. Rapid urban expansion caused other LULC types to change. Accordingly, farmland area was significantly decreased by 1855 km^2^—the largest change in area among all LULC types. The inward transfer area was 456 km^2^, and the outward transfer area was 2311 km^2^. The most dominant land transition was from farmland to construction land, with an area of 1448 km^2^. To some extent, urban expansion occurred at the expense of farmland.

The impacts of 10 potential driving factors associated with social and economic conditions on LULC variation were investigated. All 10 indicators considered in this study were positively correlated with the area of construction land and negatively correlated with farmland area. In general, social and economic development contributed considerably to urban expansion and cultivated land loss. The greatest contributors were non-agricultural population and economic conditions (secondary industry output, primary industry output and local revenues). The requirements for a higher quality life caused by population growth promoted economic development and resulted in LULC change.

Relevant policies were implemented by the government to control the proportion, usage and benefit of different LULC types, causing land use transition. Between 1987 and 1996, local government required people to return reclaimed land to forests or lakes. Some farmlands were transformed into greening land (326 km^2^) or water bodies (449 km^2^). Between 1996 and 2007, the policy of returning farmland to forest was further implemented to improve ecosystem services, which enhanced the transfer of farmland to greening land (525 km^2^). Land marketization accelerated the process of urbanization and replacement of farmland by construction land (578 km^2^). Between 2007 and 2016, prime cropland protection was one of the main government-guided policies. The inward transfer area of farmland from other types significantly increased, with an area of 1069 km^2^. Simultaneously, urban expansion continued, with an inward transfer area of 906 km^2^ from other types into construction land.

Government’s guidance and policies were considered as the internal impetus for LULC transition. Socioeconomic development goals affected the demand for different land use types and related land use policies. However, social and economic development and related land use policies were characterized by stages. This study monitored the cross transformations of different LULC types in different subperiods and provided a socioeconomic explanation based on Yearbook data. To some extent, the reported results established a relationship between human activities and LULC transfer but could not explain the specific driving mechanism of socioeconomic factors with respect to mutual transfer of different LULC types during different subperiods. Therefore, further research is required to determine how social demands and land use policies in different development stages influence the cross transformation of different LULC types.

## Figures and Tables

**Table 1:** Area statistics of different LULC classes during 1987–2016 in Wuhan.

LULC Type		Farmland	Greening Land	Water Bodies	Construction Land
1987	Area (km^2^)	5943	720	1627	291
	Proportion (%)	69.26	8.39	18.96	3.39
1996	Area (km^2^)	5211	902	1929	539
	Proportion (%)	60.73	10.51	22.48	6.28
2007	Area (km^2^)	4574	1314	1522	1171
	Proportion (%)	53.30	15.31	17.74	13.65
2016	Area (km^2^)	4088	1132	1439	1922
	Proportion (%)	47.64	13.19	16.77	22.40
1987–1996	Area (km^2^)	−732	182	302	248
	Rate (%)	−12.32	25.28	18.56	85.22
1996–2007	Area (km^2^)	−637	412	−407	632
	Rate (%)	−12.22	45.68	−21.10	117.25
2007–2016	Area (km^2^)	−486	−182	−83	751
	Rate (%)	−10.63	−13.85	−5.45	64.13
1987–2016	Area (km^2^)	−1855	412	−188	1631
	Rate (%)	−31.21	57.22	−11.56	560.48

**Table 2:** Transfer matrix among different LULC types during 1987–2016 in Wuhan.

		Farmland	Greening Land	Water Bodies	Construction Land	Total
1987–1996	Farmland	-	326	449	272	1047
	Greening land	138	-	16	22	176
	Water bodies	131	23	-	22	176
	Construction land	46	9	13	-	68
	Total	315	358	478	316	1467
1996–2007	Farmland	-	525	196	578	1299
	Greening land	182	-	19	47	248
	Water bodies	386	118	-	126	630
	Construction land	94	17	8	-	119
	Total	662	660	223	751	2296
2007–2016	Farmland	-	452	197	906	1555
	Greening land	446	-	82	171	699
	Water bodies	319	15	-	79	413
	Construction land	304	50	51	-	405
	Total	1069	517	330	1156	3072
1987–2016	Farmland	-	646	217	1448	2311
	Greening land	144	-	11	104	259
	Water bodies	279	16	-	129	424
	Construction land	33	9	8	-	50
	Total	456	671	236	1681	3044

**Table 3:** Relations among different variables considered in this study.

	X_1_	X_2_	X_3_	X_4_	X_5_	X_6_	X_7_	X_8_	X_9_	X_10_
Y_1_	0.741 **	0.850 **	0.953 **	0.919 **	0.957 **	0.949 **	0.955 **	0.953 **	0.884 **	0.919 **
Y_2_	−0.953 **	−0.953 **	−0.810 **	−0.839 **	−0.802 **	−0.813 **	−0.773 **	−0.734 **	−0.918 **	−0.854 **

Note: ** Correlation is significant at the 0.01 level (two-tailed).

**Table 4:** Multiple regression models.

Dependent Variable	Type	Regression Equation	R^2^
Construction land area	Standardized equation	Y_1_ = −1.132X_4_ + 2.082X_5_	0.949
	General equation	Y_1_ = −1.492X_4_ + 0.181X_5_ + 226.816	
Farmland area	Standardized equation	Y_2_ = −1.144X_2_ + 0.218X_8_	0.929
	General equation	Y_2_ = −2.242 × 10^−5^X_2_ + 6.755 × 10^−7^X_8_ + 319.968	

Note: All regression equations passed the 5% significance test.

## Data Availability

The data presented in this study are available upon request from the corresponding author.
